# Association between self-reported snoring and hypertension among Chinese Han population aged 30–79 in Chongqing, China

**DOI:** 10.1186/s12199-020-00908-y

**Published:** 2020-12-03

**Authors:** Meng Xiao, Xiaojun Tang, Fan Zhang, Li Zhou, Xiaoqing Bu, Xiang Liu, Xianbin Ding, Zhuozhi Shen, Liling Chen, Yunyun Wu, Wenge Tang, Jingfu Qiu

**Affiliations:** 1 School of Public Health and Management, Collaborative Innovation Center of Social Risks Governance in Health, Chongqing Medical University, No.1 Yixueyuan Road, Yuzhong District, Chongqing, 400016 China; 2 Department of Epidemiology and Health Statistics, West China School of Public Health, Sichuan University, Chengdu, 610041 Sichuan China; 3 Chongqing Center for Disease Control and Prevention, Chongqing, 400042 China

**Keywords:** Self-reported snoring, Hypertension, Chinese Han population

## Abstract

**Background:**

We aim to explore the association between self-reported snoring and hypertension among adults aged 30–79 in Chongqing, China.

**Methods:**

A total of 23,342 individuals aged 30–79 were included at baseline from August 2018 to January 2019, and the final sample size for the analysis was 22,423. Face-to-face interviews and physical examinations were conducted by trained investigators. Logistic regression was performed to study age-specific and gender-specific associations between snoring and hypertension.

**Results:**

Frequent snoring was associated with the risk of hypertension for each age and gender group, and the frequency of snoring was positively correlated with the risk for hypertension. For the three age groups (< 45, 45–59, ≥ 60), compared with the non-snoring group, those who snore often had a 64.5%, 53.3%, and 24.5% increased risk of hypertension (< 45: OR = 1.65, 95%CI 1.34–2.02; 45–59: OR = 1.53, 95%CI 1.37–1.72; ≥ 60: OR = 1.25, 95%CI 1.09–1.42), respectively. For men and women, those who snore often had a 46.8% and 97.2% increased risk of hypertension, respectively, than the non-snoring group (men: OR = 1.47, 95%CI 1.33–1.63; women: OR = 1.97, 95%CI 1.75–2.23).

**Conclusions:**

People who snore frequently should pay close attention to their blood pressure levels in order to achieve early prevention of hypertension, particularly for snorers who are female and aged under 45; importance should be attached to their blood pressure control.

## Background

Hypertension is a major risk factor for many diseases and is related to life-years lost [1, 2]. As the prevalence of hypertension is increasing in China, it introduces heavy socio-economic burden to both individuals and the health systems [3]. Hypertension control has been considered as a cost-effective way to reduce premature deaths of cardiovascular diseases (CVD) in some developing countries, like Cuba [4]. Snoring is common in population [5], which is the result of increasing resistance of the upper airway during sleep. The air flow through the narrow part of the upper airway vibrates the airway wall and surrounding soft tissues, leading to a loud snoring sound. Previous epidemiological studies have demonstrated that snoring is associated with CVD [6–8], and it was considered to be a predictor of hypertension independently of sleep apnea [9], which may play a role in the prevention and control of hypertension. However, limited attention has been paid to the association between snoring and hypertension in Southwest China. Health behaviors such as smoking, drinking, and exercise have often been taken into account when exploring the risk factors of hypertension [10], while some other factors, including snoring, have been overlooked by researchers. This study investigated whether self-reported snoring was associated with hypertension in a large-scale Chinese Han population in Chongqing, China, and whether this association, if any, differ by age and gender.

## Methods

### Study participants and design

**Fig. 1:**
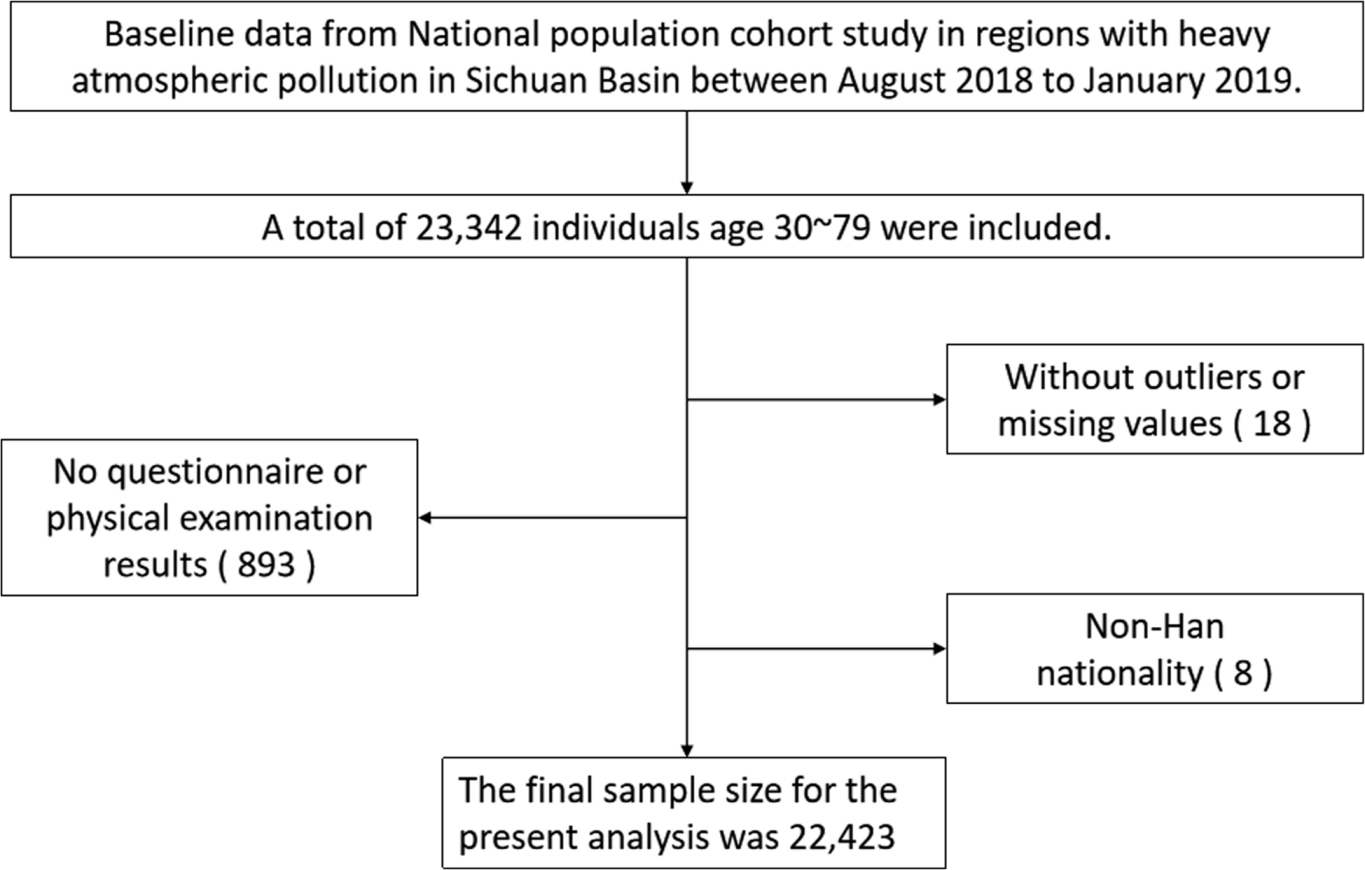
Data cleaning flow chart

We used data from the baseline survey of the China Multi-Ethnic Cohort Study (Chongqing region). Twenty thousand Chinese Han adults were planned to be recruited in Chongqing in this prospective cohort study. Since September 2018, this population-based survey was carried out in 13 districts/counties by randomized sampling (the district and county are of the same administrative level in Chongqing), including Yuzhong District, Jiulongpo District, Nan’an District, Ba’nan District, Changshou District, Jiangjin District, Hechuan District, Qijiang District, Dazu District, Tongnan District, Rongchang District, Wulong District, and Fengdu County. The number of targeted participants in each district/county was allocated according to the age and gender structure of Chongqing population in 2018. Individuals were enrolled on the basis of the following criteria: (1) 30–79 years old (i.e., born between January 1, 1939, and December 31, 1988); (2) household registered in Chongqing; (3) Han ethnicity; (4) residence for half a year or more in the local area; (5) voluntarily participate in the survey, agree to collect biological samples, and sign an informed consent; and (6) no mental illness or other related diseases, the ability of expression and understanding is normal. Individuals were excluded if having the following conditions: (1) those with severe disability who cannot do the physical examination, (2) those who do not agree to participate in the whole study, (3) those who do not give consent to be followed up, and (4) those who are not willing to be registered by real name. Twenty-three thousand people were expected to be included in the initial sampling, and a total of 23,342 individuals aged 30–79 were recruited at baseline from August 2018 to January 2019. Among them, 23,044 individuals completed physical examinations at baseline. In the present study, we analyzed the data of both the complete questionnaires and the related physical examination results. Therefore, the final sample size for the present analysis was 22,423 (Fig. 1). Written informed consent forms were obtained from all participants. This study was approved by the Ethics Committee of Sichuan University (No. K2016038).

### Data collection

Face-to-face interviews and physical examinations were conducted by investigators who were trained based on the standard study protocols before the baseline survey commenced (including how to complete the electronic questionnaires using pads which record the interviews during the survey, how to check and upload the questionnaires, standards of anthropometric measurements, collection and handover requirements of blood and urine specimens). The electronic questionnaires were conducted with the consent of the participants, and qualified questionnaires were uploaded by the investigators after checking. Recordings were randomly checked by trained post-graduates from Sichuan University for quality control every day. Unqualified questionnaires were re-investigated. If the investigator was found unqualified several times, he/she was replaced. Blood and urine samples were tested by a third-party laboratory (Chongqing Di’an Medical Laboratory Center Limited).

### Data analysis

The explanatory variables in our study included age, sex, marital status, education level, family history of hypertension, self-reported smoking, drinking, physical activity, nocturnal sleep duration (hours), snoring, midday nap duration (minutes), body mass index (BMI), abdominal obesity, dyslipidemia, and blood pressure. Age was grouped as per a commonly used age group stratification model in China [11], in which people aged between 45 and 59 years old were defined as middle-aged people. Marital status was grouped into “married/cohabitated” or “other” (separated/divorced/widowed/never married). Education level was grouped into “primary school or below,” “junior middle school,” and “high school or above” (based on the highest degree completed). Smoking was grouped into “ever,” “never,” and “current.” Drinking and physical activity were both grouped into “never or hardly,” “occasionally or sometimes,” and “often or every day.” Nocturnal sleep duration was grouped into “< 7 h,” “7 h,” “8–9 h,” and “> 9 h” according to National Sleep Foundation’s sleep duration recommendation [12]. Self-reported snoring was ascertained by the question “Do you snore when you have a sleep?” Those who reported “yes” were further asked about the frequency of snoring. Accordingly, participants were grouped into “no,” “sometimes,” and “frequently.” Midday nap duration was grouped as follows: “0 min,” “< 60 min,” and “≥ 60 min” [13, 14]. BMI was calculated using the height and weight values recorded during the physical examination; it was grouped into “< 24,” “24–27.9,” and “≥ 28” on the basis of previous studies since those whose BMI was less than 18.5 at baseline were few in the current study [15]. Men with a waist circumference (WC) ≥ 85 cm or women with a waist circumference ≥ 80 cm were regarded as abdominal obesity, which was defined based on Chinese guidelines on the prevention and control of obesity [16]. Participants were regarded as having dyslipidemia if any of the following four items were met: serum total cholesterol (TC) level of ≥ 6.22 mmol/l, triacylglycerol (TG) level of ≥ 2.3 mmol/l, low-density lipoprotein cholesterol (LDLC) level ≥ 4.1 mmol/l, and high-density lipoprotein cholesterol (HDLC) level < 1.0 mmol/l [17]. Blood pressure in the right arm was measured in a resting condition three times consecutively (1 min apart). Hypertension was defined as a systolic or diastolic blood pressure (SBP/DBP) of ≥ 140/90 mmHg or a history of hypertension diagnosed by doctors [18].

The data were analyzed using the Statistical Analysis System (SAS) 9.4 (SAS Institute Inc., Cary, NC, USA). Categorical variables were described by frequency and percentage, while continuous variables were showed by median (Q1, Q3), chi-square test, and Wilcoxon rank sum test that were performed in the univariate analysis. Logistic regression was performed to study the association between self-reported snoring and hypertension. The level of significance was set at a two-sided *p* < 0.05.

## Results

### Characteristics of participants by groups of self-reported snoring

The characteristics of participants based on groups of self-reported snoring are presented in Table 1. Of the 22,423 participants, half of them snored. Participants snoring were more likely to be aged 45–59 and men, and among those who were “married/cohabitation,” “education level of high school or above,” “no family history of hypertension,” “never smoking,” “never or hardly drinking,” “never or hardly taking part in physical exercise,” “no midday nap duration,” and “sleep 8~9 h at night” groups, snoring was less common. Compared with participants who do not snore, snorers had a higher level of SBP and DBP (*P* < 0.05), and higher proportion of them had BMI over 24, abdominal obesity, and dyslipidemia (*P* < 0.05).

**Table 1:** Characteristics of participants by groups of self-reported snoring

Variables	No	Sometimes	Frequently	***H***/***χ***^**2**^	***P***
***N*** **(%)**	11,484 (51.2)	5990 (26.7)	4949 (22.17)		
**Socio-demographic characteristics**					
Age (year)					
< 45	4140 (36.1)	1800 (30.1)	977 (19.7)	*χ*^2^ *=* 505.34	< 0.01
45–59	4686 (40.8)	2629 (43.9)	2235 (45.2)		
> 59	2658 (23.2)	1561 (26.1)	1737 (35.1)		
Sex					
Male	4052 (35.3)	3177 (53.0)	3271 (66.1)	*χ*^2^ *=* 1445.20	< 0.01
Female	7432 (64.7)	2813 (47.0)	1678 (33.9)		
Marital status					
Married/cohabitated	10,076 (87.7)	5278 (88.1)	4358 (88.1)	*χ*^2^ *=* 0.65	0.72
Others	1408 (12.3)	712 (11.9)	591 (11.9)		
Education level					
Primary school or below	3810 (33.2)	1711 (28.6)	1826 (36.9)	*χ*^2^ *=* 120.90	< 0.01
Junior middle school	3719 (32.4)	1902 (31.7)	1592 (32.2)		
High school or above	3955 (34.4)	2377 (39.7)	1531 (30.9)		
Family history of hypertension					
No	7812 (68.0)	3654 (61.0)	(59.5)	*χ*^2^ *=* 147.27	< 0.01
Yes	3672 (32.0)	2336 (39.0)	2004 (40.5)		
**Health-related behavioral characteristics**					
Smoking					
Ever	508 (4.4)	426 (7.1)	459 (9.3)	*χ*^2^ *=* 857.93	< 0.01
Never	9299 (81.0)	4149 (69.3)	2956 (59.7)		
Current	1677 (14.6)	1415 (23.6)	1534 (31.0)		
Drinking					
Never or hardly	6086 (53.0)	2300 (38.4)	1873 (37.9)	*χ*^2^ *=* 732.68	< 0.01
Occasionally or sometimes	3982 (34.7)	2462 (41.1)	1746 (35.3)		
Often or every day	1416 (12.3)	1228 (20.5)	1330 (26.9)		
Physical activity					
Never or hardly	4973 (43.3)	2265 (37.8)	2244 (45.3)	*χ*^2^ *=* 105.55	< 0.01
Occasionally or sometimes	2367 (20.6)	1276 (21.3)	812 (16.4)		
Often or every day	4144 (36.1)	2449 (40.9)	1893 (38.3)		
Midday nap duration (min)					
= 0	5154 (44.9)	2367 (39.5)	2001 (40.4)	*χ*^2^ *=* 97.59	< 0.01
< 60	3149 (27.4)	1843 (30.8)	1288 (26.0)		
≥ 60	3181 (27.7)	1780 (29.7)	1660 (33.5)		
Nocturnal sleep duration (h)					
< 7	3150 (27.4)	1863 (31.1)	1705 (34.5)	*χ*^2^ *=* 132.69	< 0.01
7 < 8	3127 (27.2)	1743 (29.1)	1387 (28.0)		
8–9	4736 (41.2)	2207 (36.8)	1683 (34.0)		
> 9	471 (4.1)	177 (3.0)	174 (3.5)		
**Clinical variables**					
SBP (mmHg)^a^	124.3 (113.7, 138.3)	129.0 (118.3, 142.0)	134.3 (123.0, 148.3)	*H* = 896.82	< 0.01
DBP (mmHg)^a^	76.0 (69.3, 83.3)	78.67 (71.7, 86.7)	82.0 (74.3, 89.7)	*H* = 866.19	< 0.01
BMI (kg/m^2^)					
< 24	6334 (55.2)	2356 (39.3)	1202 (24.3)	*χ*^2^ *=* 1786.77	< 0.01
24–27.9	4105 (35.8)	2736 (45.7)	2360 (47.7)		
≥ 28	1045 (9.1)	898 (15.0)	1387 (28.0)		
Abdominal obesity					
No	6255 (54.5)	2462 (41.1)	1306 (26.4)	*χ*^2^ *=* 1145.84	< 0.01
Yes	5229 (45.5)	3528 (58.9)	3643 (73.6)		
Dyslipidemia					
No	9197 (80.1)	4332 (72.3)	3176 (64.2)	*χ*^2^ *=* 481.29	< 0.01
Yes	2287 (19.9)	1658 (27.7)	1773 (35.8)		

^a^These continuous variables were non-normality, represented by median (Q1, Q3), and the comparison among groups was performed by Kruskal-Wallis *H* test

Prevalence of hypertension among participants with different snoring frequency in different age and gender groups is displayed in Table 2. In the three age groups, those who reported “snore frequently” were more likely to have hypertension than other groups (*P* < 0.05). In the comparison results of different genders, higher prevalence of hypertension was also found in the “snore frequently” group (*P* < 0.05).

**Table 2:** Prevalence of hypertension among participants with different snoring frequency

Snoring	Age [***n*** (%)]			Sex [***n*** (%)]	
	< 45	45–59	> 59	Male	Female
**No**	386 (9.3)	1336 (28.5)	1506 (56.7)	1371 (33.8)	1857 (25.0)
**Sometimes**	316 (17.6)	934 (35.5)	921 (59.0)	1205 (37.9)	966 (34.3)
**Frequently**	290 (29.7)	1080 (48.3)	1134 (65.3)	1625 (49.7)	879 (52.4)
***χ***^**2**^	287.17	261.13	33.06	197.51	498.59
***P***	< 0.01	< 0.01	< 0.01	< 0.01	< 0.01

Table 3 shows the results of logistic regression by self-reported snoring frequency: the odds ratio (OR) and 95% confidence interval (CI) for hypertension. Variables showed statistical significance (*P* < 0.05) in the univariate analysis was brought into the logistic regression. For all age and gender groups, those who reported “snore frequently” had a higher odds of hypertension in all models (*P* < 0.05) compared to those who do not snore.

**Table 3:** Logistic regression of the association between self-reported snoring frequency and hypertension

Variables	Sometimes		Frequently		Likelihood ratio		Wald	
	OR (95%CI)	***P***	OR (95%CI)	***P***	***χ***^**2**^	***P***	***χ***^**2**^	***P***
**Age**								
**< 45**								
Model 1	2.07 (1.76, 2.43)	**< 0.01**	4.11 (3.45, 4.88)	**< 0.01**	260.02	**< 0.01**	264.69	**< 0.01**
Model 2	1.65 (1.39, 1.95)	**< 0.01**	2.74 (2.27, 3.30)	**< 0.01**	468.85	**< 0.01**	433.73	**< 0.01**
Model 3	1.66 (1.40, 1.97)	**< 0.01**	2.74 (2.26, 3.31)	**< 0.01**	483.99	**< 0.01**	445.60	**< 0.01**
Model 4	1.33 (1.11, 1.59)	**< 0.01**	1.65 (1.34, 2.02)	**< 0.01**	737.73	**< 0.01**	635.48	**< 0.01**
**45~59**								
Model 1	1.38 (1.25, 1.53)	**< 0.01**	2.35 (2.11, 2.60)	**< 0.01**	256.80		255.81	
Model 2	1.29 (1.16, 1.43)	**< 0.01**	2.05 (1.84, 2.29)	**< 0.01**	444.97	**< 0.01**	425.66	**< 0.01**
Model 3	1.30 (1.17, 1.44)	**< 0.01**	2.05 (1.84, 2.28)	**< 0.01**	507.05	**< 0.01**	479.50	**< 0.01**
Model 4	1.14 (1.02, 1.27)	**0.02**	1.53 (1.37, 1.72)	**< 0.01**	923.91	**< 0.01**	823.38	**< 0.01**
**> 59**								
Model 1	1.10 (0.97, 1.25)	0.14	1.44 (1.27, 1.63)	**< 0.01**	33.35	**< 0.01**	32.93	**< 0.01**
Model 2	1.10 (0.97, 1.26)	0.14	1.45 (1.28, 1.65)	**< 0.01**	169.49	**< 0.01**	162.20	**< 0.01**
Model 3	1.13 (0.99, 1.27)	0.07	1.48 (1.30, 1.68)	**< 0.01**	219.34	**< 0.01**	208.08	**< 0.01**
Model 4	1.06 (0.93, 1.21)	0.39	1.25 (1.09, 1.42)	**< 0.01**	405.65	**< 0.01**	372.66	**< 0.01**
**Sex**								
**Male**								
Model 5	1.20 (1.09, 1.32)	**< 0.01**	1.93 (1.76, 2.12)	**< 0.01**	196.34	**< 0.01**	195.42	**< 0.01**
Model 6	1.22 (1.11, 1.35)	**< 0.01**	1.88 (1.70, 2.07)	**< 0.01**	538.92	**< 0.01**	508.91	**< 0.01**
Model 7	1.21 (1.10, 1.34)	**< 0.01**	1.86 (1.68, 2.05)	**< 0.01**	785.76	**< 0.01**	714.82	**< 0.01**
Model 8	1.10 (0.99, 1.22)	0.08	1.47 (1.33, 1.63)	**< 0.01**	1125.65	**< 0.01**	976.82	**< 0.01**
**Female**								
Model 5	1.57 (1.43, 1.73)	**< 0.01**	3.30 (2.96, 3.68)	**< 0.01**	474.69	**< 0.01**	473.82	**< 0.01**
Model 6	1.56 (1.42, 1.72)	**< 0.01**	2.76 (2.46, 3.09)	**< 0.01**	1324.13	**< 0.01**	1158.36	**< 0.01**
Model 7	1.57 (1.42, 1.74)	**< 0.01**	2.70 (2.40, 3.03)	**< 0.01**	1614.76	**< 0.01**	1347.21	**< 0.01**
Model 8	1.36 (1.23, 1.51)	**< 0.01**	1.97 (1.75, 2.23)	**< 0.01**	2151.47	**< 0.01**	1693.47	**< 0.01**

“No snoring” was taken as the reference group

Model 1: unadjusted model

Model 2: adjusted for sex, education level, and family history of hypertension

Model 3: adjusted for sex, education level, family history of hypertension, smoking, drinking, midday nap duration, and nocturnal sleep duration

Model 4: adjusted for sex, education level, family history of hypertension, smoking, drinking, midday nap duration, nocturnal sleep duration, BMI, abdominal obesity, and dyslipidemia

Model 5: unadjusted model

Model 6: adjusted for age, education level, and family history of hypertension

Model 7: adjusted for age, education level, family history of hypertension, smoking, drinking, midday nap duration, and nocturnal sleep duration

Model 8: adjusted for age, education level, family history of hypertension, smoking, drinking, midday nap duration, nocturnal sleep duration, BMI, abdominal obesity, and dyslipidemia

## Discussion

Previous studies have found that frequent snoring may be positively associated with the risk of acute myocardial infarction [19] and colorectal cancer [20]. In the present study, we found that frequent snoring increased the risk of hypertension in each age and gender group. After further adjusting for the confounders, there was still a strong association between snoring and hypertension, and snoring frequency was positively correlated with the risk for hypertension.

Our findings are consistent with those from previous studies. An analysis of 580 adults [5] found an increased risk of hypertension and CVD associated with snoring, which shared views similar to our results. Sossai et al. [21] enrolled 202 subjects from Limana in 2014, and they verified the link between snoring and hypertension in their observational study. They found a significant relationship between snoring and hypertension. This may be because snoring was relevant to increased sympathetic tone and consequent arterial hypertension. Some researchers believe that snoring may reduce sleep quality [22], while poor sleep quality was related to hypertension [23] and may even increase the risk of hypertension [24]. Adrian et al. [25] believe exposure to snoring may cause larger left ventricles in one’s hearts, which makes the heart wall thicker and the heart contract harder. This may explain the association between snoring and blood pressure to some extent.

Compared with previous studies, we conducted a more detailed analysis which was stratified by age and gender. It seems that the risk decreases with age, and for the three age groups (< 45, 45–59, ≥ 60), compared with non-snoring group, those who snore often had a 64.5%, 53.3%, and 24.5% increased risk of hypertension [OR = 1.65, 95%CI (1.34–2.02); OR = 1.53, 95%CI (1.37–1.72); OR = 1.25, 95%CI (1.09~1.42)], respectively. Snoring was found to have an important effect on hypertension, particularly, in people under 45 years old. Hypertensive patients tend to be younger in recent years [26], people under 45 who snore often should not neglect the changes in their blood pressure. In this study, for men and women, those who snore often had a 46.8% and 97.2% increased risk of hypertension than the non-snoring group [OR = 1.468, 95%CI (1.33–1.63); OR = 1.97, 95%CI (1.75–2.23)], respectively. A new report [27] showed that women’s hearts were more vulnerable to snoring than men’s_._ As mentioned above, snoring may have an impact on the heart and thus the blood pressure, suggesting that snoring women should pay more attention to their heart and blood pressure. Hu et al. [28] carried out a survey which enrolled 73,231 American women, after adjustment for age, BMI, WC, and other covariates, and found that snoring was still associated with higher prevalence of hypertension, as well as higher systolic and diastolic blood pressure levels. In addition, both theirs and our results suggested people snored more often was associated with a significantly higher risk of hypertension in women after adjustment. However, Hu et al. [28] only conducted the survey among women and only those aged 40–65 years were enrolled. In this study, our data also showed frequent snoring also increased the risk of hypertension in men before we adjusted for BMI, abdominal obesity, and dyslipidemia, although the difference between those non-snoring and occasionally snoring was not found in men after adjusting for all confounders. This implies that for men, BMI, abdominal obesity, or dyslipidemia may affect the relationship between occasionally snoring and hypertension. Besides, the difference in results of men and women indicated the necessity to stratify the results by gender. Since hypertension was probably an expression of metabolic disease, the results of the clinical indicators may have an impact on snoring, which may affect the relationship between snoring and hypertension. Zhang et al. [29] found self-reported snoring to be significantly associated with dyslipidemia, especially high TC and high LDLC. Troxel et al. [30] believe that loud snoring may indicate the development of metabolic syndrome based on their results from a prospective study with a 3-year follow-up. These results also showed the necessity of adjusting the confounding factors like clinical indicators.

In addition, our results suggested that in Chongqing, almost half of people aged 30–79 snore when they sleep, and there is a male predominance in prevalence, which has been confirmed in a previous meta-analysis [31], and Physiological differences such as the differences in upper airway anatomy, and hormonal influences may explain the gender difference. Snoring appears to be more common among smokers. Franklin et al. [32] found that even passive smoking is an underlying risk factor for snoring, and alcohol may have an immediate effect on snoring. A previous study [33] showed that physical activity may exert a protection on snoring, which suggested the importance of good lifestyles.

Snoring is regarded as a major symptom of obstructive sleep apnea (OSA) syndrome. According to previous studies [27, 34, 35], the high activity of sympathetic nerve, the increase of circulating catecholamine caused by it, and the increase of vasoconstrictor sensitivity may be the mechanisms linking OSA to blood pressure. Some studies [36, 37] have suggested that vibration induced by snoring is related to repeated local Inflammation and can promote the release of interleukin-8 in the airway inflammatory cell model, which are considered to be the possible local inflammatory effects in the pathogenesis of hypertension. However, the underlying mechanisms of snoring on hypertension need further exploration.

## Strength and limitation

Snoring is widely diffused in population. Previously, some studies have been carried out to investigate the relationship between snoring and hypertension in other countries [21, 38]. However, to our knowledge, this study was the first one to explore the relationship between self-reported snoring and hypertension by using the cross-sectional data of a large-scale cohort study in Chongqing, which is one of four municipalities that are located in southwest China with a population of more than 30 million [39]. Despite some previous studies have focused on the association between snoring and hypertension, limited attention has been paid to the differences between genders and age groups. Taking the influence of age and gender on the association between snoring and hypertension into consideration [40–42], we conducted this analysis stratifying by age and sex, and we comprehensively considered the related confounders to explore the relationship between snoring and hypertension among Chinese people in this study. Different ethnic backgrounds may influence the results of the study [43]; therefore, our study only included the Han ethnicity in China, which may not be taken into account in other studies. In addition, large samples and strict quality control increased the representativeness and generalizability of our data to some extent. However, this study has several limitations. We only analyzed cross-sectional data from the cohort study; it can only explain whether self-reported snoring frequency was related to hypertension, and the casual relationship could not be examined. Further longitudinal data and specific modeling approaches are needed to determine the causal relationship between snoring and hypertension. Our study only took the subjective frequency of snoring into consideration while the frequency was not quantified and the degree of snoring was ignored, which may have an impact on the results, and future studies need to take this into account. The snoring was self-reported by participants, not measured through objective measurements, so there may be a bias between self-reported and actual snoring. However, a highly significant association between self-reported snoring and snoring objectively recorded by microphone has been found in a previous study [44], and the sensitivity and specificity were 94% and 58%, respectively, suggesting that self-reported snoring is representative despite its limitations. There was an age limit in our study population, so caution should be taken when extrapolating the results to other age groups. Besides, considering the change of hormone in women before and after menopause should be taken into account in further studies.

## Conclusions

Frequent snoring was associated with hypertension in all age and gender groups, and people who snoring frequently should pay close attention to their blood pressure levels in order to achieve early prevention of hypertension, especially for snorers under the age of 45 and snoring women; importance should be attached to their blood pressure control.

## Data Availability

The data that support the findings of this study cannot be shared publicly because of ethical restrictions imposed by the institutional ethics committee, as the data contain sensitive information on participants. However, the data can be made available from the corresponding author for all interested researchers upon requests sent to the author’s office. The initial contact for request should be addressed to the corresponding author’s institution.

## Abbreviations

CVD: Cardiovascular diseases
BMI: Body mass index
WC: Waist circumference
TC: Total cholesterol
TG: Triacylglycerol
LDLC: Low-density lipoprotein cholesterol
HDLC: High-density lipoprotein cholesterol
SBP: Systolic blood pressure
DBP: Diastolic blood pressure
SAS: Statistical Analysis System
